# Supplementary material for: Zika Virus Infection Induces Interleukin-1β-Mediated Inflammatory Responses by Macrophages in the Brain of an Adult Mouse Model
Source: J Virol. 2023 May 16;97(6):e00556-23. doi: 10.1128/jvi.00556-23 (PMC10308908; doi:10.1128/jvi.00556-23)
Supplement: Supplemental file 1 — Table S1. Download jvi.00556-23-s0001.docx, DOCX file, 0.01 MB [file jvi.00556-23-s0001.docx]

Table S1. The sequences of qPCR primers and probes used in this study.

| Gene | Primer 1 (5’-3’) | Primer 2 (5’-3’) | Probe (5’-3’) |
| --- | --- | --- | --- |
| ZIKV | CACGCTTGGAACAAACCAAA | TTATGGACACCGAAGTGGAAG | 56-FAM/TCAGGCTTT/ZEN/GATTGGGTGACGGAT/3IABkFQ |
| Il1b | GACCTGTTCTTTGAAGTTGACG | CTCTTGTTGATGTGCTGCTG | 56-FAM/TTCCAAACC/ZEN/TTTGACCTGGGCTGT/3IABkFQ |
| Il6 | AGCCAGAGTCCTTCAGAGA | TCCTTAGCCACTCCTTCTGT | 56-FAM/CCTACCCCA/ZEN/ATTTCCAATGCTCTCCT/3IABkFQ |
| Ifng | CTGAGACAATGAACGCTACACA | TCCACATCTATGCCACTTGAG | 56-FAM/TCTTGGCTT/ZEN/TGCAGCTCTTCCTCA/3IABkFQ |
| Tnf | AGACCCTCACACTCAGATCA | TCTTTGAGATCCATGCCGTTG | 56-FAM/CCACGTCGT/ZEN/AGCAAACCACCAAGT/3IABkFQ |
| Cebpb | GTTTCGGGACTTGATGCAATC | CCGCAGGAACATCTTTAAGTGA | 56-FAM/ACACGGGAC/ZEN/TGACGCAACACA/3IABkFQ |
| C3 | CCTTCCACCTTTTTCCTTCACT | CTCCAGCCGTAGGACATTG | 56-FAM/AGGGTCCCA/ZEN/GCTACTAGTGCTACTG/3IABkFQ |
| Actb | GATTACTGCTCTGGCTCCTAG | GACTCATCGTACTCCTGCTTG | 56-FAM/CTGGCCTCA/ZEN/CTGTCCACCTTCC/3IABkFQ |
